# Zoonotic Disease Management and Infection Control Practices Among Veterinarians in the United Arab Emirates

**DOI:** 10.3390/vetsci8050082

**Published:** 2021-05-11

**Authors:** Ihab Habib, Zainab Alshehhi

**Affiliations:** 1 Department of Veterinary Medicine, College of Food and Agriculture, United Arab of Emirates University, Al Ain P.O. Box 1555, United Arab Emirates; 201402254@uaeu.ac.ae; 2 School of Veterinary Medicine, The College of Science, Health, Engineering and Education, Murdoch University, Perth 6150, Australia

**Keywords:** zoonoses, infection prevention, veterinary public health, occupational health

## Abstract

This study was conducted to assess zoonotic disease management and infection control practices (ICPs) among veterinarians in the United Arab Emirates (UAE). A questionnaire was developed in SurveyMonkey, an online tool, and was distributed by email during February–May 2020 to 470 veterinarians practicing across the UAE. A total of 110 individuals completed the survey, giving a response rate of 23.4% (110/470). Results indicate that reported hand hygiene, sharps management, barrier or isolation practices, and personal choices for personal protective equipment (PPE) in common practice scenarios varied among practitioners. The majority (>75%) of veterinarians in all practice types reported always washing their hands before eating, drinking, or smoking at work. The survey revealed that 19% and 10% of large and small animal veterinarians indicated they sterilized and reused disposable needles. Veterinarians among all practices indicated high rates (75% to 80%) of recapping needles before disposal. When handling an animal suspected of having a zoonotic disease, most (90%) of small animal veterinarians reported always using practices such as isolating the animal and removing outwear before contact with other animals. However, only half (55%) of the large animal respondents reported always isolating the animal or sterilizing all equipment used on the animal of concern. Fewer than half of the large animal (35%) and mixed practice (44%) veterinarians indicated they would always be limiting human contact with the animal of concern. All of the small animal respondents reported full compliance with PPE while performing surgery and necropsy. Among large animal veterinarians, 44% reported not using respiratory or eye protection when aiding with parturition or handling conception products. Failure to use appropriate PPE when handling blood samples was the second most common noncompliant practice among large animal (39%) veterinarians and mixed practice (41%) respondents. Our study indicates a need for continuous education regarding ICPs in the veterinary community in the UAE. Better awareness of the risk of zoonotic disease exposure and options for managing this risk and liability issues could drive the adoption of infection control practices.

## 1. Introduction

Zoonoses are diseases and infections that are naturally transmitted between vertebrate animals and humans. Approximately 60% of all human pathogens are zoonotic; all types of potential pathogenic agents, including bacteria, viruses, parasites, and fungi, can cause these zoonotic infections [1]. Zoonotic diseases can cause endemic disease with long-term social and economic impacts or be featured as acute outbreaks leading to high morbidity and mortality. Professionals working in animal health and husbandry have inherently high risks of exposure to and infection with zoonotic diseases [2,3]. It has been reported that around 30–40% of the veterinarians surveyed in the USA reported having been infected with zoonotic diseases [4]. In Australia, around 50% of the veterinarians (*n* = 344) reported contracting a zoonosis during their careers, of which 25.2% reported a true incidence [5]. Worldwide, veterinarians and animal health staff are estimated to file almost 3-times more occupational health claims than human-health workers [6].

Veterinarians are trained to help prevent the transmission of zoonotic diseases by recognizing and treating diseases in companion and food animals; these professionals play an essential role in promoting public health by educating clients about disease transmission from animals to humans [7]. Veterinary knowledge of zoonotic disease prevention and suspected case management is crucial—veterinarians are at the frontline of defense against zoonoses’ entry into the human population [8]. In the United Arab Emirates (UAE), a five-year veterinary medicine program was launched in 2013, and the first-ever batch of Emirati veterinary doctors was graduated in 2018 from the United Arab Emirates University (UAEU) [9]. The UAE is home to one of the world’s highest immigrant populations, and it is not surprising to find wide variability in educational backgrounds and training experiences among the veterinary workforce in UAE, which is dominated by non-Emirati professionals [10]. The internationalization of the veterinary workforce in the UAE is a unique feature that adds value to the profession’s strength; however, it also implies some pressing challenges. There is a need for adopting standardized infection control practices (ICPs) in veterinary medicine practices across the UAE to prevent zoonotic disease transmission. UAE federal authorities have developed a range of preventative practices guidelines (e.g., UAE Ministry of Climate Change and Environment) and individual agencies at the Emirate levels (e.g., The Abu Dhabi Agriculture and Food Safety Authority) to promote biosecurity in the animal health work environment [11,12]. The effectiveness of any guidelines depends on their uptake by animal health practitioners. Nevertheless, to date, we know very little about veterinarians’ perception and adoption of zoonoses management practices and ICPs in the UAE.

There has been no published research that looked at ICPs and zoonoses risk management perceptions of veterinarians in the UAE. In recent years, outbreaks of Middle East respiratory syndrome and Crimean-Congo hemorrhagic fever have shown a critical challenge arising at the human–animal interface in the UAE [13,14]. Therefore, the purpose of this study was to evaluate a subset of practicing veterinarians in the UAE to assess the knowledge, attitudes, and practices concerning ICPs as well as to facilitate an understanding of self-perceived zoonotic disease risk in veterinary practice.

## 2. Material and Methods

### 2.1. Study Design and Respondent Recruitment

The study was designed as a cross-sectional survey with the target population defined as English-speaking practicing veterinarians registered to work in the UAE. A practicing veterinarian was defined as a nationally registered veterinarian who treats diseases, disorders, and injuries in animals in a clinical setting [15]. Thus, in the scope of this study, veterinarians working in slaughterhouses, diagnostic laboratories, and other non-clinical settings were not included.

A comprehensive list of 500 practicing veterinarians’ contact details was supplied by a veterinary pharmaceutical distributor company working in the UAE; the company has a regularly updated business list of practicing veterinary clinics across all the UAE. The study was part of senior (fifth-year veterinary student) project course work and was reviewed and examined by a committee from the Department of Veterinary Medicine of the UAEU.

### 2.2. Survey Implementation

The survey instrument was refined from previous research to address the present research objectives [5,16]. The questionnaire was then piloted with five veterinary professionals, and after reviewing the feedback, the survey was further refined. The final questionnaire was designed through the web-based application SurveyMonkey (https://www.surveymonkey.com/, accessed on 25 October 2020). The survey link was distributed by email, followed by four reminders, during February–May 2020. Basic demographic information was collected, and the identities of the respondents were anonymous. The study proposal was approved by the social science research ethics committee of UAEU (ERS_2019_5775). Participants were provided with an information sheet concerning the study’s background and were requested to sign the consent form before completing the web-based survey. The questionnaire was designed to assess respondent practices regarding ICPs and zoonosis case management by utilizing a 24-variable tool that was divided into three sections (Table 1): Basic hygienic behavior at the workplace (6 questions); measures that are taken when a suspect animal has a zoonotic disease (4 questions); and personal protective equipment (PPE) adoption in specific situations (10 questions) [16]. The suitability of PPE’s level to specific scenarios was based on guidelines of the National Association of State (the United States of America) Public Health Veterinarians [17]. 

Responses on veterinarian’s adoption of hygienic behavior at the workplace and measures taken when a suspect animal has zoonotic disease were based on a five-point scale (never, seldom, sometimes mostly, and always). For PPE compliance in specific animal handling situations, the respondents were asked to choose one of five categorical answers reflecting their choice of personal protective equipment: (1) no special precautions are taken, (2) protective clothes or gloves only, (3) protective clothing and gloves, (4) gloves, protective clothing, and mask or face shield, and (5) gloves, protective clothing, plus mask and face shield [16,17].

### 2.3. Data Management and Analysis 

We computed simple descriptive statistics (frequencies and percentages) for each survey question using tools embedded in the SurveyMonkey platform. To determine whether gender (male versus female) and age (≥45 years versus <45 years) were associated with inadequate infection control practices (ICPs), respondents were compared based on calculating a precaution awareness score (PA score) for each category [16]. A score of 0 through 4 was assigned to each response related to the categories and situations listed in Table 1. Higher scores were assigned as respondents reported behaviors more likely to protect against zoonotic disease transmission or use of additional PPE when handling animals or engaging in specific activities. Scores were summed for each individual (PA score). Within each practice type, respondents were categorized based on their PA scores in the upper 25% or lower 75% of summed scores (designated as high or low PA rankings, respectively) [16]. A low PA score corresponded to less than ideal ICPs. Univariate logistic regression analysis was performed to compare the two categories of respondents (male vs. female; ≥45 years vs. <45 years) according to their PA score (high vs. low PA score). Data were analyzed using STATA version.15; a *p*-value of <0.05 was considered statistically significant. 

## 3. Results

### 3.1. Respondents’ Characteristics

Of the 500 listed veterinarians, 470 had valid email addresses. A total of 110 individuals completed the survey, giving a response rate of 23.4% (110/470). Clinic characteristics and demographics of the respondents by practice type were assessed (Table 2). Most veterinarians working in small animal practice were female, but large animal and mixed practice veterinarians were predominantly male—half of the respondents working in small animal practice aged ≥45 years. The majority (80%) of small animal veterinarians reported a work practice experience of ≥10 years. None of the small animal veterinarians and few large animals and mixed practice veterinarians reported having board certification (Table 2).

### 3.2. Hygienic Behavior at Workplace

Table 3 provides veterinarians’ responses to the frequency of behavior related to hand hygiene, sharp management, and isolation practices. The majority (>75%) of veterinarians in all practice types reported always washing their hands before eating, drinking, or smoking at work. Few (5%) large animal veterinarians reported mostly eating or drinking in the animal handling area. Large animal veterinarians appeared less likely to wash or sanitize hands between patient contacts, with around half (52%) of the respondents reporting they always engage in this behavior. 

Veterinarians among all practices reported high rates (75% to 80%) of recapping needles before disposal. Nevertheless, 10% of small animals (*n* = 20) and mixed practice veterinarians (*n* = 40) indicated that they never apply such a critical practice (Table 3). Only 76% (*n* = 42) of large animal veterinarians indicated that they always used an approved container to dispose of used sharps, compared with 90% and 95% of small animals and mixed practice veterinarians, respectively. Some veterinarians from each practice type reported sterilizing and using disposable needles or syringes intended for one-time use (Table 3). It is worth noting that the practice of reusing disposable needles was more common among large animal veterinarians than in small animal and mixed practice respondents. When handling an animal suspected of having a zoonotic disease, most (90%) of small animal veterinarians reported always using practices such as isolating the animal and removing outwear before contact with other animals. Around half (55%) of the large animal respondents reported always isolating the animal or sterilizing all equipment used on the animal of concern. Fewer than half of the large animal (35%) and mixed practice (44%) veterinarians indicated they would always be limiting human contact with the animal of concern (Table 3).

### 3.3. Assessment of Infection Control Practices (ICPs)

**Figure 1:**
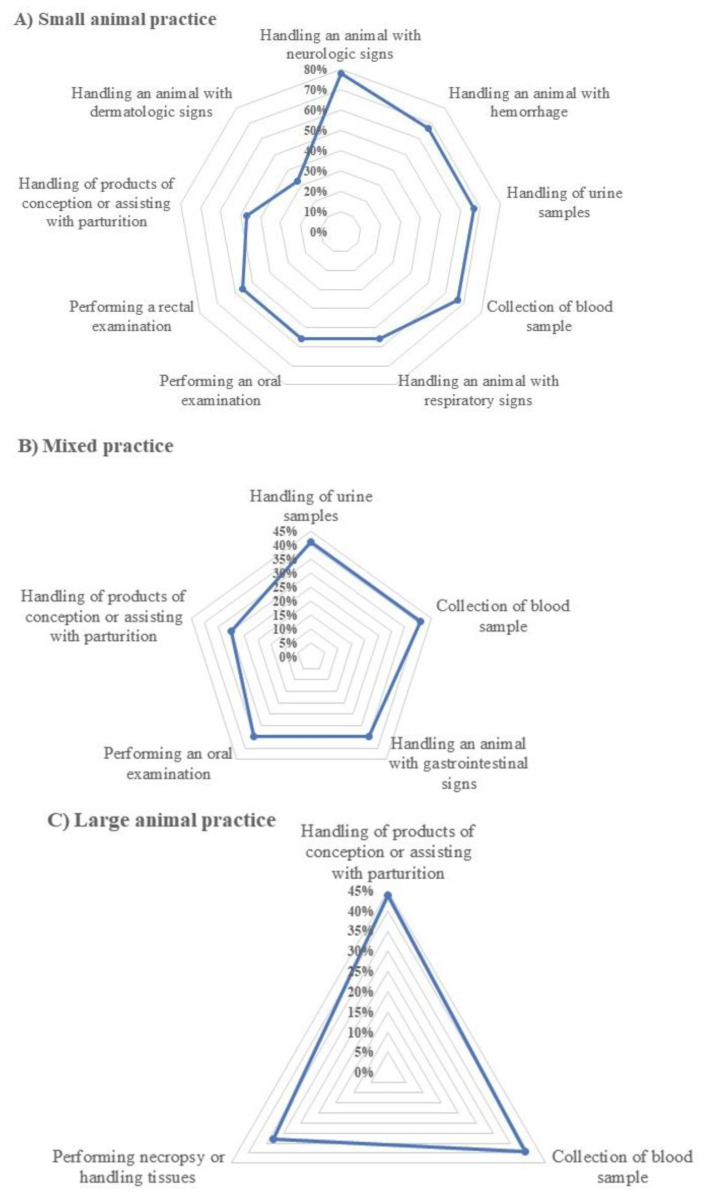
Commonly overlooked (>30%) PPE compliance practices in clinical activities, as reported by veterinarians in the United Arab Emirates, 2020. For each practice type, a two-dimensional radar chart plots a series of values (overlooked practices) over multiple quantitative variables (%, frequency). Each variable has its own axis; all axes are joined in the center of the figure.

The reported adoption level of PPE by veterinarians for selected practice scenarios was also assessed (Table 4). Veterinarians from each practice type chose to use some combination of PPE more frequently when examining a healthy animal than when examining an ill animal. However, appropriate PPE was used by <75% of small animal veterinarians when examining animals with dermatologic (67%), respiratory (44%), gastrointestinal (44%), or neurologic signs (22%). All of the small animal respondents reported full compliance with PPE while performing surgery and necropsy. Among large animal veterinarians, 44% failed to use respiratory or eye protection when aiding with parturition or handling conception products (Figure 1). Failure to use appropriate PPE when handling blood samples was the second most common noncompliant practice among large animal (39%) veterinarians as well as among mixed practice (41%) respondents (Figure 1).

### 3.4. Association of Gender and Age with Infection Control Practices (ICPs)

In each practice type, the precaution awareness score (PA score) was calculated based on the responses received from the respondents. Veterinarians’ low PA score rankings were compared with data for those with high PA scores to determine whether gender and age characteristics were associated with less adoption of ICP (Table 5). Analysis using logistic regression revealed no association between age and PA scores; however, there was an evident association between gender (male vs. female) and lower PA scores in small and large animal clinics. Being a male was associated with higher odds (Odds Ratio (OR) = 1.83 (95% CI, 1.30 to 2.85; *p* < 0.001)) of low PA score among the surveyed small animal veterinarians. For large animal veterinarians, the analysis revealed that being a male was significantly associated with lower PA scores (OR, 1.93; 95% CI, 1.06 to 3.53; *p* = 0.03).

## 4. Discussion

This study evaluated infection control practices applied by veterinarians and identified some aspects for enhancing further safety and biosecurity measures in veterinary practices in the United Arab Emirates. Results indicate that reported hand hygiene, sharps management, barrier or isolation practices, and personal choices for PPE in common practice scenarios varied among practitioners. For instance, our results reveal that most (75%−80%) practitioners reported always washing hands before eating or drinking in animal handling areas and washing or sanitizing hands between patient contacts. Compared with our finding, a lower rate of handwashing practice was reported in a survey in the United States, where 55.2% of small animal veterinarians (*n* = 1069) reported always washing their hands before eating and drinking at work, and 48.4% (516/1066) reported always washing their hands between patient contacts [16]. Unwashed hands pose a risk for nosocomial transmission among veterinary patients, as well as a risk for zoonotic disease transmission to humans [18]. The more appropriate practice could be promoted by adopting infection control policies requiring handwashing and by designating staff rooms or eating areas separate from animal handling areas in clinic settings [19]. Our results indicate that hand washing practices in the veterinary clinic setting need to improve, with the overall goal of 100% compliance as recommended by previous studies in the United States [15,16].

In the present study, respondents always indicated sterilizing and reusing disposable needles among 19% and 10% of large and small animal veterinarians, respectively. Such reported practice has been noted in previous studies assessing infection control in veterinary practices [16,20], however, with a much lower frequency than our finding. Wright et al. (2008) reported that fewer than 1% of small animal veterinarians and fewer than 2% of large animal veterinarians surveyed in the USA are engaged with sterilizing and reusing disposable needles [16]. Practices such as washing and reusing needles and syringes present an unacceptable and preventable risk for parenteral exposures to pathogens in blood samples [20]. Single-use needles have been widely adopted in infection control guidelines in human medicine to enhance protection against bloodborne pathogens. Similar concerns should be widely recognized by veterinarians practicing in the UAE, where few but critical bloodborne zoonotic pathogens (e.g., Crimean-Congo Hemorrhagic Fever and Rift Valley Fever viruses) have been reported in the past and are potentially transmitted through contact with blood, including needle injections [13]. Education is useful in improving compliance with workplace safety guidelines that recommend never recapping needles among human healthcare providers and may be similarly useful in promoting appropriate ICP among veterinary practitioners. 

Veterinarians who do not consider PPE in their routine work are compromising their duty of care to adopt work practices that do not expose themselves, their supporting team, and others to avoidable risk of zoonotic diseases [5]. Despite that a high proportion of respondents in this study indicated excellent PPE adoption practices while performing surgery, there is room for improved veterinary work culture practices in the UAE. For instance, 78% of the respondents working in small animal practice indicated handling an animal with neurologic signs without particular precaution, protective clothes, or gloves. Previous research has identified animal-related injuries, particularly dog and cat bites, as essential hazards in the veterinary profession [21]. The most frequent biological hazard for western Canadian veterinarians was animal bites, with or without accompanying infections [22]. Bites and scratches were injuries that contributed to workdays lost by staff in an Australian study [23]. Safe animal handling practices may reduce the risk from all biological hazards (e.g., reducing infection with Rabies and Cat Scratch Disease) but specifically could reduce the number of animal bites. 

On the other hand, for large animal veterinarians involved in this study, the most common (among 55% of the respondents) risky practice was handling products of conception or assisting with parturition without wearing protective clothing and gloves plus surgical mask, goggles, or face shield. In the UAE, although animal (sheep, goats, camels) surveillance data are sparse, there is likely a significant animal reservoir of Brucella infection [24]. Across the Eastern Mediterranean region, sheep, goats, camels, and their products are the primary infection sources. Consequently, brucellosis has been an occupational risk for farmers, veterinary surgeons, and employees in the meatpacking business. Veterinarians should be well informed that small droplets or aerosols of body fluids (with associated high risk for exposure to several agents that can be involved during parturition, for example, *Brucella* spp. and *Coxiella burnetii* transmission) can be released during both procedures and carry a risk for zoonotic diseases exposure through contact with splashes or droplets of the body fluids [25].

Based on the present study findings (Table 5), gender significantly influences the difference in infection control practices (ICPs) between small and large animal veterinarians. Logistic regression analysis of the received responses revealed that small animal and large animal veterinarians who were male were significantly more likely to have low precaution awareness scores (PA score), indicating less than ideal ICPs. Our finding is comparable to that noted by Wright et al. [16], where male veterinarians also reported lower compliance with ICPs than female veterinarians in the USA. Added to that, results of previous self-reported studies indicate that in regular community settings and even healthcare settings, males may be less likely to comply with handwashing recommendations than females [26,27]. Findings in our study indicated that gender is associated with differences in veterinarians’ approaches to ICPs; thus, educational initiatives that are tailored toward men may be warranted.

The results of the study and this report are subject to limitations. First, the sample included may not fully represent the broader population of veterinarians registered in the UAE. For instance, we received only one complete survey from an avian and wildlife veterinarian, which obviously does not represent well this professional group. Although it is not rare to select a nonrandom sample for conducting veterinary observational studies, we acknowledge such an impact on the study’s external validity [5]. Despite our efforts in sending multiple reminders, we ended up with a response rate of 23.4% (110/470). This might have been attributed to the fact that this survey addressed a potentially sensitive topic (i.e., personal choices regarding infection control). It could be speculated that the demographics of veterinarians who responded may have been different from those who chose not to participate, which might introduce a source of bias to the study results.

## 5. Conclusions

Our study indicated a need for education and policy regarding ICPs in the veterinary profession in the UAE. Better awareness of the risk of zoonotic disease exposure and options to manage this risk and liability issues could drive the adoption of infection control practices. Targeted education of UAE practicing veterinarians about zoonotic disease risks is needed, along with a widespread campaign to increase awareness amongst veterinarians of the guidelines and standards developed by the competent UAE authorities relating to biosecurity and infection control programs, as well as their liabilities and legal responsibilities. Further study is required to understand the reasons for differences in compliance with PPE use in different practice types in the UAE context.

## Figures and Tables

**Table 1:** The survey instrument used to assess zoonosis case management and infection control practices of practicing veterinarians—the United Arab Emirates, 2020. The survey instrument was adapted based on Dowd et al. [5] and Wright et al. [16].

**Hygienic Behavior at the Workplace**
(1) Washing hands before eating, drinking, or smoking at work
(2) Eating or drinking in animal handling areas
(3) Washing or sanitizing hands between patient contacts
(4) Recapping of needles before disposal
(5) Disposal of needles in an approved sharps container
(6) Sterilization and reuse of disposable needles or syringes
**Measures Taken when a Suspect Animal has a Zoonotic Disease**
(7) Isolation or quarantine of the animal
(8) Restriction of No. of people that have contact with the animal
(9) Removal of outerwear before contact with other animals
(10) Sterilization of all equipment after use on the animal
**Personal Protective Equipment (PPE) Compliance in Specific Situations**
(11) Handling a healthy animal
(12) Handling an animal with dermatologic signs
(13) Handling an animal with respiratory signs
(14) Handling an animal with gastrointestinal signs
(15) Handling an animal with neurologic signs
(16) Handling an animal with hemorrhage
(17) Handling of fecal samples
(18) Handling of urine samples
(19) Handling of products of conception or assisting with parturition
(20) Collection of a blood sample
(21) Performing an oral examination
(22) Performing a rectal examination
(23) Performing surgery
(24) Performing necropsy or handling tissues

**Table 2:** Demographics and practice characteristics of survey respondents—the United Arab Emirates, 2020.

	Practice Type		
Variable	Large Animal (*n* = 48)	Small Animal (*n* = 20)	Mixed Practice (*n* = 42)
**Demographic**			
Male	36 (75%)	6 (30%)	30 (71.4%)
Age ≥45 years	12 (25%)	10 (50%)	6 (14.3%)
Practicing veterinary medicine ≥10 years	24 (50%)	16 (80%)	22 (52.3%)
Owner or partner in practice	4 (8%)	12 (60%)	2 (4.7%)
Working ≥40 h/week	38 (79.1%)	8 (40%)	34 (80.9%)
Board certification	6 (12.5%)	-	6 (14.2%)
**Practice characteristic**			
Teaching/referral hospital	8 (16.6%)	-	4 (9%)
Mobile services only	4 (8.3%)	-	2 (5%)
Clinic services only	12 (25%)	12 (60%)	18 (43%)
Clinic and mobile services	22 (45.8%)	8 (40%)	18 (43%)

**Table 3:** Self-reported hygienic behavior at workplace among veterinarians, by practice type—the United Arab Emirates, 2020.

Variables Related to Hygienic Behavior at the Workplace	Practice Type (No. of Respondents)	Reported Frequency				
		Never	Seldom	Sometimes	Mostly	Always
Washing hands before eating, drinking, or smoking at work	LA (*n* = 42)	0%	0%	5%	19%	76%
	SA (*n* = 20)	0%	0%	0%	20%	80%
	MIXED (*n* = 40)	0%	0%	5%	20%	75%
Eating or drinking in animal handling areas	LA (*n* = 42)	48%	33%	14%	**5%**	0%
	SA (*n* = 20)	50%	30%	20%	0%	0%
	MIXED (*n* = 40)	70%	25%	5%	0%	0%
Washing or sanitizing hands between patient contacts	LA (*n* = 42)	0%	**10%**	10%	28%	52%
	SA (*n* = 20)	0%	0%	0%	10%	90%
	MIXED (*n* = 40)	0%	0%	5%	35%	60%
Recapping of needles before disposal	LA (*n* = 42)	0%	0%	5%	19%	76%
	SA (*n* = 20)	**10%**	**10%**	0%	0%	80%
	MIXED (*n* = 40)	**10%**	0%	5%	10%	75%
Disposal of needles in an approved sharps container	LA (*n* = 42)	0%	0%	14%	10%	76%
	SA (*n* = 20)	**10%**	0%	0%	0%	90%
	MIXED (*n* = 40)	0%	0%	0%	5%	95%
Sterilization and reuse of disposable needles or syringes	LA (*n* = 42)	67%	0%	9%	**5%**	**19%**
	SA (*n* = 20)	90%	0%	0%	0%	**10%**
	MIXED (*n* = 40)	75%	5%	5%	**10%**	**5%**
Isolation or quarantine of the animal	LA (*n =* 40)	0%	**5%**	30	10%	55
	SA (*n* = 20)	0%	0%	0%	10%	90
	MIXED (*n* = 36)	0%	0%	11%	17%	72%
Restriction of No. of people that have contact with the animal	LA (*n* = 40)	0%	**15%**	45%	5%	35%
	SA (*n* = 20)	0%	0%	0%	30%	70%
	MIXED (*n* = 36)	0%	0%	28%	28%	44%
Removal of outerwear before contact with other animals	LA (*n* = 40)	0%	**5%**	25%	10%	60%
	SA (*n* = 20)	0%	**10%**	0%	0%	90%
	MIXED (*n* = 36)	0%	**5%**	17%	22%	56%
Sterilization of all equipment after use on the animal	LA (*n* = 40)	0%	0%	10%	35%	55%
	SA (*n* = 20)	0%	0%	0%	20%	80%
	MIXED (*n* = 36)	0%	0%	5%	17%	78%

Large animal practice (LA); Small animal practice (SA); Mixed practice (MIXED).

**Table 4:** Self-reported compliance with personal protective equipment (PPE) in specific professional situations among veterinarians, by practice type—the United Arab Emirates, 2020.

Professional Activity	Practice Type (No. of Respondents)	* Levels of Personal Protective Equipment (PPE)			
		Level 1	Level 2	Level 3	Level 4
Handling a healthy animal	LA (*n* = 36)	6%	22%	44%	28%
	SA (*n* = 18)	22%	56%	11%	11%
	MIXED (*n* = 34)	12%	18%	47%	23%
Handling an animal with dermatologic signs	LA (*n* = 36)	0%	22%	39%	39%
	SA (*n* = 18)	0%	33%	45%	22%
	MIXED (*n* = 34)	0%	9%	18%	53%
Handling an animal with respiratory signs	LA (*n* = 36)	0%	17%	28%	55%
	SA (*n* = 18)	11%	45%	11%	33%
	MIXED (*n* = 34)	6%	23%	18%	53%
Handling an animal with gastrointestinal signs	LA (*n* = 36)	0%	22%	33%	45%
	SA (*n* = 18)	22%	34%	22%	22%
	MIXED (*n* = 34)	0%	35%	30%	35%
Handling an animal with neurologic signs	LA (*n* = 36)	0%	11%	33%	56%
	SA (*n* = 18)	22%	56%	22%	0%
	MIXED (*n* = 34)	6%	23%	18%	53%
Handling an animal with hemorrhage	LA (*n* = 36)	0%	28%	22%	50%
	SA (*n* = 18)	11%	56%	22%	11%
	MIXED (*n* = 34)	0%	23%	18%	59%
Handling of urine samples	LA (*n* = 36)	0%	22%	33%	45%
	SA (*n* = 18)	11%	56%	22%	11%
	MIXED (*n* = 34)	0%	41%	24%	35%
Collection of a blood sample	LA (*n* = 36)	6%	33%	33%	28%
	SA (*n* = 18)	22%	45%	22%	11%
	MIXED (*n* = 34)	6%	35%	24%	35%
Performing an oral examination	LA (*n* = 36)	0%	22%	22%	56%
	SA (*n* = 18)	11%	45%	22%	22%
	MIXED (*n* = 34)	0%	35%	18%	47%
Performing a rectal examination	LA (*n* = 36)	0%	29%	29%	42%
	SA (*n* = 18)	0%	56%	33%	11%
	MIXED (*n* = 34)	0%	18%	23%	59%
Handling of products of conception or assisting with parturition	LA (*n* = 36)	0%	11%	33%	56%
	SA (*n* = 18)	11%	33%	11%	45%
	MIXED (*n* = 34)	0%	24%	6%	70%
Performing surgery	LA (*n* = 36)	0%	0%	22%	78%
	SA (*n* = 18)	0%	0%	0%	100%
	MIXED (*n* = 34)	0%	6%	6%	88%
Performing necropsy or handling tissues	LA (*n* = 36)	0%	11%	22%	67%
	SA (*n* = 18)	0%	0%	0%	100%
	MIXED (*n* = 34)	0%	6%	12%	82%

* Level 1, No particular precaution; **Level 2**, Protective clothing or gloves; **Level 3**, Protective clothing and gloves; **Level 4**, Protective clothing and gloves plus a surgical mask, goggles, or face shield. Based on guidelines of the National Association of State Public Health Veterinarians. Compendium of Veterinary Standard Precautions: zoonotic disease prevention in veterinary personnel, 2015. Available at: http://www.nasphv.org/documentsCompendiaVet.html, accessed on 25 October 2020

**Table 5:** Age and gender association with precaution awareness score (PA score) of survey respondents—the United Arab Emirates, 2020. Within each practice type, respondents were categorized according to their PA scores as being in the upper 25% or lower 75% of summed scores (designated as high or low PA ranking, respectively). Data are presented as % of veterinarians. The PA score categorization was adapted based Wright et al. [16].

Variable	Practice Type					
	Large Animal		Small Animal		Mixed Practice	
	Low PA Score	High PA Score	Low PA Score	High PA Score	Low PA Score	High PA Score
Age ≥ 45 years	58.2%	54.7%	53.7%	49.5%	47.8%	49.1%
Male gender	87.2% *	74.3%	55.2% *	36.3%	53.1%	52.9%

* Variable associated with low PA score (univariate logistic regression, *p* < 0.05).

## Data Availability

The data used to support this study’s findings are included within the article.
